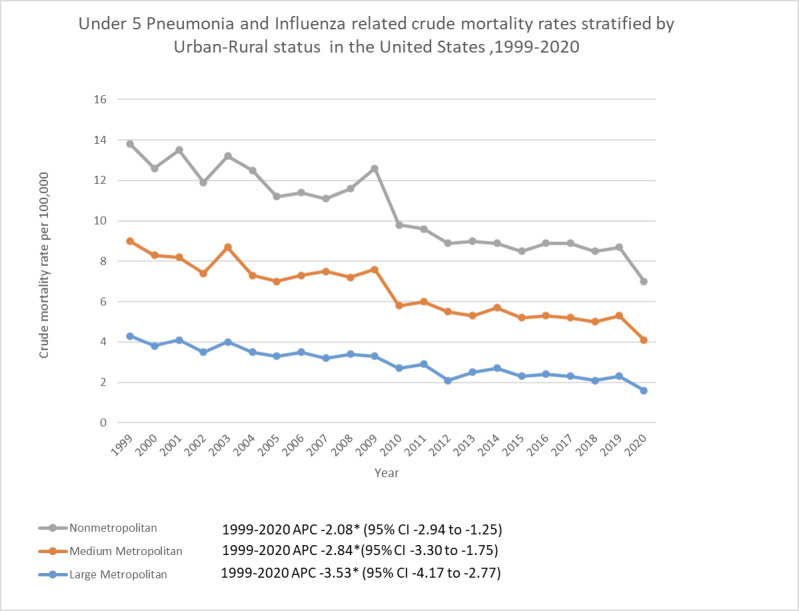# Emerging age, sex, ethnoracial, and regional trends in pneumonia and influenza-related mortality among children from 1999 to 2020

**DOI:** 10.1097/MD.0000000000042027

**Published:** 2025-04-25

**Authors:** Rubyisha Sheikh, Nushma Shaikh, Mateen Ahmed, Zara Jamil, Maria Camp Wala, Kashish Khan, Biya Maqsood, Eiman Zeeshan, Priyanka Keshav Lal, Muhammad Taha Khan, Fatima Ali Raza, Ajeet Singh, Mohamed Daoud, Samia Aziz Sulaiman

**Affiliations:** a Department of Internal Medicine, Karachi Medical and Dental College, Karachi, Pakistan; b Department of Internal Medicine, Hamdard University, Delhi, India; c Department of Internal Medicine, Allama Iqbal Medical College, Lahore, Pakistan; d Department of Internal Medicine, Dow International Medical College, Karachi, Pakistan; e Department of Internal Medicine, Dow Medical College, Karachi, Pakistan; f Department of Internal Medicine, Baqai Medical University, Karachi, Pakistan; g Department of Dentistry, Dow Dental College, Karachi, Pakistan; h Department of Internal Medicine, Dow University of Health Sciences, Karachi, Pakistan; i Department of Internal Medicine, Bogomolets National Medical University, Kyiv, Ukraine; j School of Medicine, University of Jordan, Amman, Jordan.

**Keywords:** influenza, mortality trends, pneumonia

## Abstract

Deaths related to pneumonia and influenza have been consistently declining overall in the United States (US). However, pneumonia remains one of the highest causes for morbidity and mortality, demographic and regional trends and disparities in pneumonia and influenza-related mortality must be comprehensively studied. This study analyzed mortality data extracted from the CDC WONDER database from 1999 to 2020 for children under 5 years of age. Crude mortality rates (CMRs) were calculated and Joinpoint regression analysis was used to identify trends based on annual percentage changes (APCs) values. A total of 17,229 pneumonia and influenza-related deaths occurred among children < 5 years between 1999 and 2020 (CMR: 3.3; 95% CI: 3.2–3.3). CMRs were consistently higher in male children (CMR: 3.5; 95% CI: 3.5–3.6) and among Black Americans (CMR: 5.8; 95% CI: 5.6–6.0), while lowest in Asian/Pacific Islanders (CMR: 2.4; 95% CI: 2.2–2.6). Among states, CMRs were highest in Alaska (CMR 6.9; 95% CI: 5.6–8.5). Nonmetropolitan areas had comparatively higher CMRs (CMR: 4; 95% CI: 2.9–3). A consistent decline was found in pneumonia and influenza-related mortality in children < 5 years old. Targeted strategies addressing the existing disparities can help optimize health outcomes and improve survival rates in populations at risk.

## 1. Introduction

Globally, acute lower respiratory infections (ALRIs) attributed to viral and bacterial infections in the lungs and airways constitute a significant cause of morbidity and mortality in children.^[1,2]^ Moreover, characterized by its acute impact on the lower respiratory tract, pneumonia, which is often caused by pathogens entering the lower respiratory tract below the larynx through various means - such as inhalation or aspiration -, remains the primary cause of pediatric mortality worldwide in children under 5.^[3,4]^ Pneumonia is one of the most commonly observed complications in hospitalized children with the influenza virus, considered another infectious agent that raises the burden of respiratory illnesses in children worldwide.^[5,6]^

The global incidence of pneumonia in children under 5 shows significant variation, with rates estimated at 0.05 episodes per child year in industrialized nations and 0.29 episodes per child year in underdeveloped regions.^[7]^ Streptococcus pneumonia and respiratory syncytial virus infections are linked to over 25% and 18.3% of all severe respiratory infection episodes in young children worldwide.^[1]^ With newborns accounting for 2% of these deaths, this discrepancy translates to about 2 million deaths annually for children under 5, or 15% of all childhood mortality globally.^[8,9]^ On the other side, 290,000 and 650,000 respiratory deaths are linked to the roughly 1 billion seasonal influenza episodes that occur each year, including 3 to 5 million instances of severe disease.^[10]^

Though the incidence and fatality rates of pediatric influenza and pneumonia have declined globally, national and international coordination of efforts is still essential. To keep lowering the burden of death from influenza and pneumonia in children under 5, these programs concentrate on tackling particular risk factors and demographic groupings. Populations at most significant risk can be identified using demographic and regional distribution data, enabling prompt and focused interventions. Thus, from 1999 to 2020, our goal was to investigate regional and demographic differences in the mortality linked to influenza and pneumonia in children under 5 in the United States (US).

## 2. Materials and methodology

### 2.1. Patient eligibility and screening

Deaths occurring in the US for patients under 5 were sourced from the Centers for Disease Control and Prevention’s Wide-Ranging Online Data for Epidemiologic Research (CDC WONDER) database, which covers data from 1999 to 2020. We utilized the International Classification of Diseases Tenth Revision (ICD-10) codes for pneumonia and influenza (J09–J18) to identify deaths related to pneumonia and influenza. As this study employed de-identified data that was readily available to the public, it does not require approval from an Institutional Review Board. The analysis adheres to the Strengthening the Reporting of Observational Studies in Epidemiology (STROBE) guidelines to ensure methodological precision.

### 2.2. Data abstraction and synthesis

The data were stratified by year, sex, race/ethnicity, age group, urban-rural classification, and state. Single age groups delineated age groups, including ages < 5 years. Race/ethnicity was categorized into Asian/Pacific Islanders, Black Americans, White Americans, and Hispanic/Latinos. The data extracted for the American Indian/Alaskan Native population was irrelevant regarding pneumonia and influenza. The population was split into large and medium metropolitans and nonmetropolitan sectors using the National Center for Health Statistics Urban-Rural Classification Scheme 2013.^[11]^

The CMR per 100,000 individuals was calculated by dividing the number of pneumonia and influenza-related deaths over the total population each year. AAMRs were not applicable as we used single-year age groups. CMRs were utilized to analyze deaths stratified by sex, race/ethnicity, urban or rural areas, and all states, including the District of Columbia. The average annual percentage changes and annual percentage changes (APCs) in crude rates and 95% CI were determined using the Joinpoint Regression Program 5.0.2.^[12]^ The best-fitting Joinpoint model was determined by examining data patterns and assessing the statistical significance of new joinpoints using Monte Carlo Permutation Tests. The APCs were categorized as ascending or descending based on whether the slope of mortality change significantly deviated from zero, with a *P*-value < .05 indicating statistical significance. The abstraction and synthesis was performed in line with previous published studies utilizing the CDC WONDER database.^[13–18]^

## 3. Results

Between 1999 and 2020, a total of 17,229 pneumonia and influenza-related deaths occurred among children aged up to 5 years. Our findings demonstrate an overall decrease in the number of deaths. For example, in 1999, the overall CMR was 2.001 (95% CI: 1.81–2.18), and in 2020, the overall CMR was the lowest, that is, 0.94 (95% CI: 0.82–1.07), suggesting improvement in the outcomes and hence with time improvement in health care provided has been achieved. However, many disparities were found in this trend in some years; for instance, the highest overall trend was seen in the year 2003 with a CMR of 2.16 (95% CI: 1.97–2.34), raising suspicion about the poor outcomes and high mortality rates. Similarly, another aberrant rise was noticed in the overall declining CMR trend in 2009 at 1.74 (95% CI: 1.57–1.90). A steep decline was observed in APC, with −2.85 (95% CI: −3.69 to −2.07) (Table S1, Supplemental Digital Content, https://links.lww.com/MD/O727, Fig. S1, Supplemental Digital Content, https://links.lww.com/MD/O725).

### 3.1. Annual mortality trends

**Figure 1.**
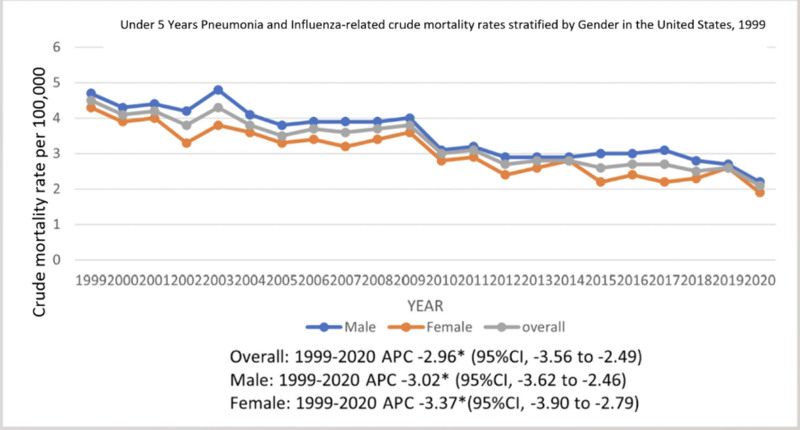
Trends in under 5 years pneumonia and influenza-related mortality overall and stratified by sex in the US, 1999–2020. * indicates the statistically significant difference of APC from 0 at α = 0.05. APC = annual percentage change, US = United States.

The crude rate for pneumonia and influenza-related deaths in children up to 5 years of age was 4.5 in 1999 which decreased to 2.1 in 2020. The overall crude rate declined from 1999 to 2020 (APC: −2.97; 95% CI: −3.56 to −2.49) (Table S2, Supplemental Digital Content, https://links.lww.com/MD/O728; Fig. 1).

### 3.2. Sex stratification

During the study period from 1999 to 2020, male children had consistently higher mortality rates than females. In 1999, the crude mortality rate for males was 4.7, which decreased to 2.2 in 2020. Similarly, the crude mortality rate for females was 4.3 in 1999 and decreased to 1.9 in 2020. The incidence of pneumonia and influenza-related deaths showed a slight decrease, with a notable reduction observed in females compared to males. The APC values for male and female children were −3.02 (95% CI: −3.62 to −2.46) and −3.37 (95% CI: −3.90 to −2.79) respectively (Table S2, Supplemental Digital Content, https://links.lww.com/MD/O728; Fig. 1).

### 3.3. Ethnoracial stratification

The CMRs for pneumonia and influenza are seen highest among Black Americans, followed by Hispanic/Latino Americans, White Americans, and Asian/Pacific Islander individuals. The crude mortality rates decreased for all the races overall from 1999 to 2020. There was a significant decline seen among Black American children. The CMR was 8.6 (95% CI: 7.7–9.5) in 1999, which dropped in 2020 to 3.5 (95% CI: 3.0–4.1) with an APC of −3.18 (95% CI: −3.9 to −2.4).

In Hispanics/Latinos, the CMR seen in 1999 was 4.4 (95% CI: 3.8–5.0), decreasing to 1.6 (95% CI: 1.3–1.9) by 2020, with a significant decline in APC: −4.15 (95% CI: −5.03 to −3.35). In the White Americans, CMR was 3.5 (95% CI: 3.3–3.8) initially in 1999, which declined by 2020 to 1.7 (95% CI: 1.5–1.9) with an APC of −2.91 (95% CI: −3.83 to −2.21).

**Figure 2.**
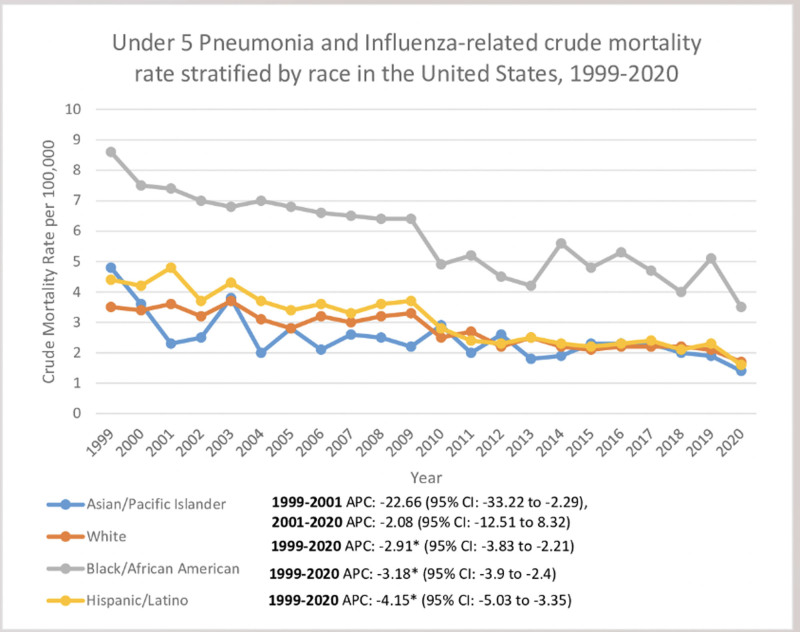
Under 5 years pneumonia and influenza-related crude mortality trends stratified by race in the US, 1999–2020. * indicates the statistically significant difference of APC from 0 at α = 0.05. APC = annual percentage change, US = United States.

In Asian/Pacific Islander children, there was a steep decrease in CMR between 1999 and 2001 from 4.8 (95% CI: 3.6–6.4) to 2.3 (95% CI: 1.4–3.4), with a remarkable APC of −22.66 (95% CI: −33.22 to −2.29). It declined in 2020 to 1.4 (95% CI: 0.9–2.1) with an APC of −2.08 (95% CI: −12.51 to 8.32) (Table S3, Supplemental Digital Content, https://links.lww.com/MD/O729; Fig. 2).

The data available on the CDC WONDER database for the American Indian/Alaska Natives was not significant; therefore, this group was excluded from the analysis.

### 3.4. State stratification

On the state-level analysis, significant disparities in crude death rates were observed. Alaska recorded the highest CMR at 6.9 (95% CI: 5.6–8.5), indicating a notable health concern in this region. Conversely, Massachusetts showed the lowest CMR at 1.5 (95% CI: 1.3–1.8), suggesting relatively better health outcomes.

States such as Louisiana (CMR: 5.7, 95% CI: 5.2–6.2), Mississippi (CMR: 5.2, 95% CI: 4.6–5.8), and Arkansas (CMR: 5.3, 95% CI: 4.7–5.9) also exhibited high crude mortality rates, aligning them with the upper percentiles of mortality rates. On the other hand, states like Connecticut (CMR: 1.5, 95% CI: 1.2–1.9) and New Jersey (CMR: 2.2, 95% CI: 1.9–2.4) were among those with lower mortality.

Geographically, the Western region had a mix of states with varying crude mortality rates. California, for example, had a CMR of 3.1 (95% CI: 2.9–3.2), which is relatively moderate. However, other Western states like Wyoming (CMR: 4.6, 95% CI: 3.3–6.1) and New Mexico (CMR: 4.5, 95% CI: 3.8–5.2) exhibited higher rates.

In the Midwest, states like Ohio (CMR: 3.7, 95% CI: 3.4–4.0) and Michigan (CMR: 3.6, 95% CI: 3.4–3.9) presented moderate crude rates, while Minnesota (CMR: 3.6, 95% CI: 3.2–4.0) showed similar trends.

The Southern region had notable extremes, with Louisiana and Mississippi at high crude mortality rates, while Virginia (CMR: 2.8, 95% CI: 2.5–3.0) and Texas (CMR: 3.3, 95% CI: 3.2–3.5) were comparatively lower but still higher than states in the Northeast.

**Figure 3.**
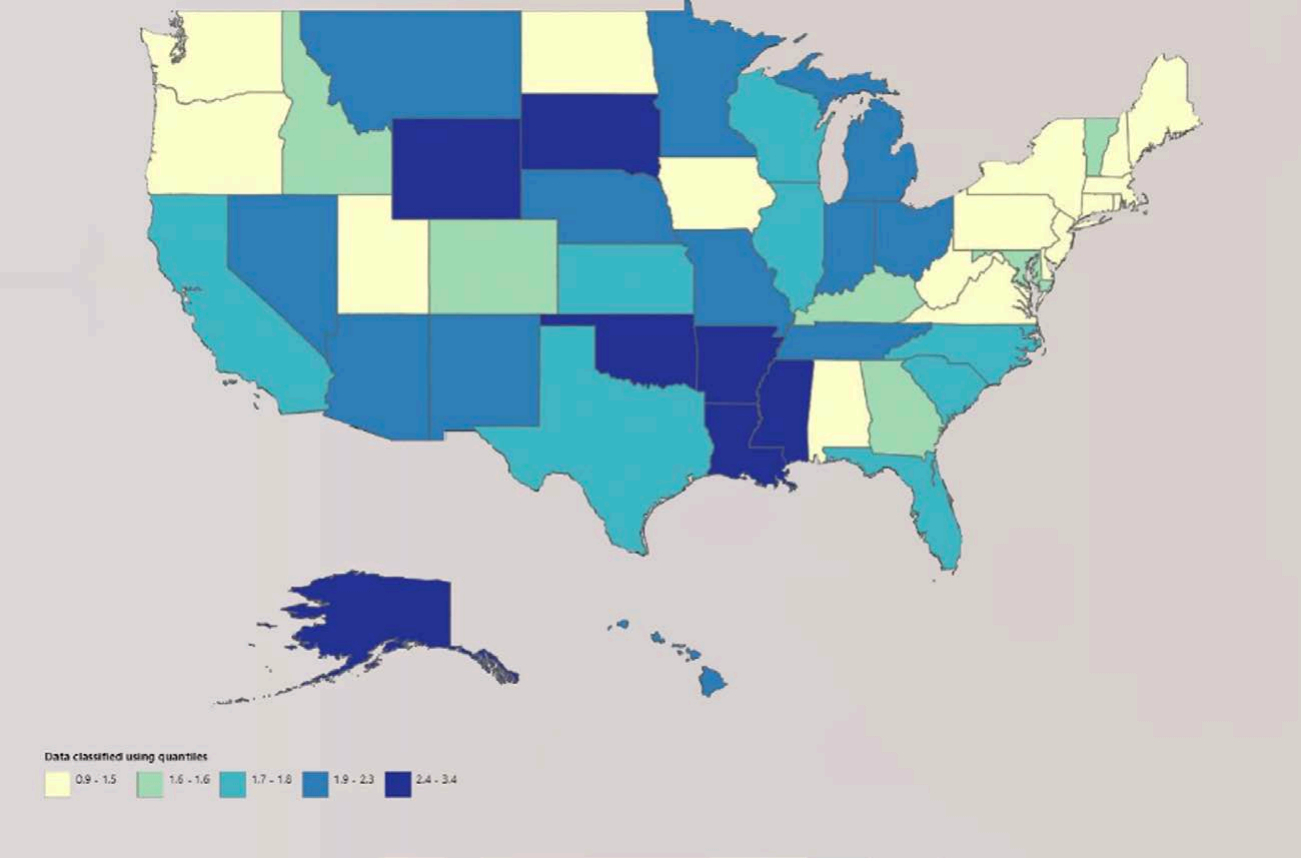
Under 5 years pneumonia and influenza-related crude mortality trends stratified by States in the US, 1999–2020. US = United States.

The Northeast region generally exhibited lower crude rates, with states like New York (CMR: 2.8, 95% CI: 2.6–3.0), Pennsylvania (CMR: 3, 95% CI: 2.8–3.2), and Massachusetts being among the lowest (Table S4, Supplemental Digital Content, https://links.lww.com/MD/O730; Fig. 3).

### 3.5. Urbanization-wise analysis

The analysis also highlighted significant disparities among the metro and nonregions in the US. Nonmetropolitan areas had comparatively higher overall CMR (CMR: 4.0; 95% CI: 2.9–3.0) than medium and large metropolitan areas with CMRs of 3.5 (95% CI: 3.4–3.6) and 3.0 (95% CI: 2.9–3.0) respectively. All the regions showed a steady drop in crude mortality rates from 1999 to 2020. Nonmetropolitan areas had an APC of −2.08 (95% CI: −2.94 to 1.25). In medium metropolitan areas, there was a notable decrease in APC (−2.48; 95% CI: −3.30 to −1.75) by 2020. Crude mortality rates decreased by a much more significant margin in large metropolitan areas in 2020, with an APC of −3.53 (95% CI: −4.17 to 2.77) (Table S5, Supplemental Digital Content, https://links.lww.com/MD/O732; Fig. S2, Supplemental Digital Content, https://links.lww.com/MD/O726).

## 4. Discussion

Our study comprehensively highlights several disparities in the mortality trends of pneumonia and influenza in children under 5 years of age over the past 2 decades. A steep decline in overall mortality from 1999 through 2020 was observed in the second decade, showing approximately half the CMR observed compared to the first. This trend remained consistent in both sexes. However, male children had a steadily higher trend compared to females. Second, Black American children exhibited the highest mortality among other racial groups. Moreover, northwest, Alaska had the highest CMR, whereas Massachusetts, in the northeast, had the lowest CMR. Significant disparities were also noted between the urban and rural regions where CMRs in medium metropolitan and nonmetropolitan areas dropped to nearly half by 2020. The CMRs in large metropolitan areas consistently declined significantly over the twenty years.

While other studies have thoroughly explored pneumonia and influenza mortality trends in the general population, to the best of the authors’ knowledge, our study is the first to explore those trends in the US pediatric population thus addressing this gap. We believe that the results of our study have significant implications for policy making within the US, and more broadly, possibly in low and middle-income countries. Implementing similar approaches could effectively reduce mortality related to pneumonia and influenza. This is supported by a meta-analysis published in 2018, which found that approximately 109.5 million cases of influenza were reported in children under 5 years old, globally. Among those cases, around 10 million developed ALRI due to the flu, leading to around 870,000 hospital admissions, 15,000 in-hospital deaths, and roughly 35,000 total deaths from influenza-related ALRI.^[2]^ According to the CDC, pneumonia and influenza rank 5th among the 15 leading causes of death in children < 5 years,^[19]^ where approximately 1.5% of children under 5 years are in a fair or poor state of health, elevating their risk of complications.^[20]^ The mortality figures related to influenza and secondary pneumonia increase during the peak season, as well as the complex interaction between individual and environmental risk factors plays a vital role in determining the outcome of disease. Children under 5 years of age, particularly those under 2 years old, and children with underlying comorbid are particularly at a higher risk of complications from influenza. A study has also found that childhood wasting, stunting, hygiene practices, immunization status, household air pollution, secondhand smoking, health insurance coverage, and judicious use of antibiotics collectively influence the outcome of pneumonia and influenza.^[21]^ Historically, Flannery et al have demonstrated that unvaccinated children 6 months of age and older accounted for up to 80% of pediatric influenza-related mortality, whereas about 70% of vaccine coverage can significantly reduce mortality in children under 5.^[22]^ Moreover, maternal influenza vaccination has been proven to minimize the risk of influenza-associated hospitalizations and deaths in infants up to 2 months of age.^[23]^ Similarly, a study conducted by Omer et al has found a 20% decrease in all-cause severe clinical pneumonia in babies under 6 months of age was shown in a pooled analysis of 3 mother influenza vaccination studies conducted in Nepal, Mali, and South Africa.^[24]^

Additionally, previous studies have shown that the existence of disparities in health status and outcomes among the different racial and ethnic populations in the US has been a concern and could be traced back to the 1918 influenza pandemic.^[25]^ The persistently higher trends in Black Americans can be attributed to multiple interacting factors that collectively contribute to the more significant burden of disease. Donaldson et al documented that such disparities may be attributed, at least in part, to socioeconomic factors such as high levels of poverty, limited availability of well-paying employment opportunities, and challenges in accessing quality healthcare and preventive services, which are more prevalent among Black Americans relative to others.^[26]^ Other factors including crowded housing and unfavorable neighborhood conditions also contribute to a more significant number of deaths in this cohort.^[27]^ However, the COVID-19 pandemic has guided policymakers into acknowledging and addressing health inequities, and the CDC has similarly declared racism a threat to public health.^[25,28]^ Initiatives like The Immunization Coalition of Los Angeles, CA, and the Metro Health Immunization Program in San Antonio, TX, focus on increasing vaccination rates and reducing inconsistencies among populations at high risk of under-immunization as highlighted by Lippert et al.^[29]^

Moreover, it must be noted that continuous assessment of vaccine safety, immunogenicity, and effectiveness is imperative, particularly for diverse and at-risk populations. Substantial efforts are currently underway to develop universal influenza vaccines that will offer broader protection and obviate the need for annual vaccinations. Additionally, understanding how immunity to influenza is established in early life and developing a vaccine safe and effective for infants below 6 months of age are crucial priorities. A study by Rolfes et al highlights the severity of the 2017 to 2018 influenza season in the US, mainly due to influenza A[H3N2] viruses. With a vaccine effectiveness of 38%, influenza vaccination significantly reduced the burden of influenza-related illness, medical visits, hospital stays, and deaths during the flu season.^[30]^ Over the last decade, the immunization status has substantially improved in the US, with 59% of children being documented to have received influenza vaccination in 2020.^[20]^ A significant proportion of children in low-income and lower-middle-income countries do not reach the age of 5 due to deaths related to influenza and pneumonia hence adopting comparable strategies could significantly reduce mortality from pneumonia and influenza in these countries.^[2]^ Our findings show significant demographic and regional variations in influenza and pneumonia mortality among children under 5 years old in the US. Targeted local interventions can be more effective in reducing mortality and illness in newborns and young children, by recognizing these differences. These insights can also be valuable for low-income countries, where adopting similar policies could greatly alleviate the disease burden. The Integrated Global Action Plan for Pneumonia and Diarrhea (GAPPD) by WHO aimed at reducing the mortality rate from pneumonia to <3 per 1000 live births by 2025.^[31]^ The Global Influenza Strategy 2019–2030 is another initiative to reduce the burden of seasonal Influenza.^[32]^ Additionally, influenza vaccination also largely contributes to reducing severe illness and mortality in children through preventing flu-associated emergency visits and hospitalizations. In a study conducted by Sumner et al on 15,728 children with acute respiratory illness, vaccination was associated with a 55.7% reduction in influenza-related ED visits and hospitalizations. The protective effect was consistent across severity levels, with vaccine effectiveness of 52.8% for ED visits, 52.3% for noncritical hospitalizations, and 50.4% for critical hospitalizations. These findings highlight the crucial role of influenza vaccines in reducing the burden of severe illness and improving survival rates in children.^[33]^

### 4.1. Limitations

There are multiple limitations in our study which could be addressed in the future. Firstly, pneumonia and influenza deaths are recorded under 1 code in the International Statistical Classification of Diseases, although they have different causes, which can result in disparities between provisional and final death counts, potentially affecting the accuracy of mortality surveillance data. Combining these classifications may thus obscure the true impact of each disease individually which complicates the development of targeted interventions. This could be improved by collecting data particularly for pneumonia-related deaths associated with influenza infection. Moreover, changes in diagnostic criteria, coding practices, and healthcare practices over time may also affect data consistency and trend analysis, which could be improved by following a standardized approach for data collection. Third, the CDC WONDER database couldn’t provide significant data for the American Indian/ Alaskan Native population. However, one can’t deny the presence of high mortality rates among this cohort.^[34]^ Fourth, data on other socioeconomic factors that affect health were also unavailable, which may influence access to care.

### 4.2. Future prospects

Our study highlights the existing disparities in pneumonia and influenza-related mortality, highlighting the urgent need to enhance healthcare access across the US and to conduct further research that explores these disparities on a global scale. Moving forward, it is essential to bridge such disparities by offering equitable healthcare delivery, ultimately leading to improved health outcomes and higher survival rates. Promoting seasonal vaccination across all demographics, particularly among populations with limited healthcare access, can facilitate the establishment of herd immunity, which is crucial to help protect high-risk individuals from complications. To further strengthen vaccination efforts, community outreach programs may be implemented to educate all populations about the importance of vaccinations and dispel myths and misinformation surrounding vaccines, thereby increasing acceptance and uptake. Collaborative efforts between public health organizations, healthcare providers, and community leaders are essential for developing such targeted public awareness campaigns that emphasize the importance of preventive measures and timely medical care. In addition, further research must be conducted to help guide healthcare systems in employing data analytics to identify high-risk populations, efficiently allocating resources, and developing community-based healthcare initiatives, which collectively pave the way towards tailored management strategies which address health inequities and contribute to mitigating the risks of deterioration.

## 5. Conclusion

In conclusion, our study shows an overall decline in mortality related to pneumonia and influenza in the US, yet highlights persistent demographic disparities which require urgent action. More thorough healthcare research is required to investigate vaccination coverage, factors associated with under-vaccination, and effective strategies to boost uptake in diverse populations. Engaging key stakeholders, including patients and families, healthcare professionals, public health officials, and community leaders, is crucial for optimizing health outcomes.

## Acknowledgments

The authors would like to acknowledge the Research Council of Pakistan (RCOP) for supporting all aspects of conducting this study.

## Author contributions

**Conceptualization:** Rubyisha Sheikh, Mateen Ahmed, Zara Jamil, Maria Camp Wala, Mohamed Daoud, Samia Aziz Sulaiman.

**Data curation:** Biya Maqsood, Priyanka Keshav Lal, Ajeet Singh.

**Formal analysis:** Rubyisha Sheikh, Nushma Shaikh, Mateen Ahmed, Ajeet Singh, Samia Aziz Sulaiman.

**Investigation:** Kashish Khan, Biya Maqsood, Muhammad Taha Khan.

**Methodology:** Nushma Shaikh, Kashish Khan, Biya Maqsood.

**Resources:** Zara Jamil, Mohamed Daoud, Samia Aziz Sulaiman.

**Software:** Eiman Zeeshan, Priyanka Keshav Lal.

**Supervision:** Rubyisha Sheikh, Samia Aziz Sulaiman.

**Validation:** Rubyisha Sheikh, Fatima Ali Raza, Mohamed Daoud.

**Visualization:** Zara Jamil, Eiman Zeeshan.

**Writing – original draft:** Rubyisha Sheikh, Nushma Shaikh, Biya Maqsood, Ajeet Singh, Mohamed Daoud, Samia Aziz Sulaiman.

**Writing – review & editing:** Muhammad Taha Khan, Fatima Ali Raza, Ajeet Singh, Samia Aziz Sulaiman.

## Supplementary Material



**Figure:**