# Supplementary material for: Emerging age, sex, ethnoracial, and regional trends in pneumonia and influenza-related mortality among children from 1999 to 2020
Source: Medicine (Baltimore). 2025 Apr 25;104(17):e42027. doi: 10.1097/MD.0000000000042027 (PMC12040052; doi:10.1097/MD.0000000000042027)
Supplement: Supplementary file 1 [file medi-104-e42027-s001.docx]

| **YEAR** | **OVERALL** | | | **MALE** | | | **FEMALE** | | |
| --- | --- | --- | --- | --- | --- | --- | --- | --- | --- |
|  | **DEATHS** | **CRUDE RATE** | | **DEATHS** | **CRUDE RATE** | | **DEATHS** | **CRUDE RATE** | |
| **1999** | 1035 | 4.5 | (4.2 - 4.7) | 551 | 4.7 | (4.3 - 5.0) | 484 | 4.3 | (3.9 - 4.7) |
| **2000** | 944 | 4.1 | (3.8 - 4.3) | 507 | 4.3 | (3.9 - 4.7) | 437 | 3.9 | (3.5 - 4.2) |
| **2001** | 983 | 4.2 | (4.0 - 4.5) | 528 | 4.4 | (4.1 - 4.8) | 455 | 4.0 | (3.6 - 4.4) |
| **2002** | 876 | 3.8 | (3.5 - 4.0) | 500 | 4.2 | (3.8 - 4.6) | 376 | 3.3 | (3.0 - 3.6) |
| **2003** | 1000 | 4.3 | (4.0 - 4.5) | 570 | 4.8 | (4.4 - 5.2) | 430 | 3.8 | (3.4 - 4.1) |
| **2004** | 903 | 3.8 | (3.6 - 4.1) | 492 | 4.1 | (3.7 - 4.4) | 411 | 3.6 | (3.2 - 3.9) |
| **2005** | 837 | 3.5 | (3.3 - 3.8) | 458 | 3.8 | (3.4 - 4.1) | 379 | 3.3 | (2.9 - 3.6) |
| **2006** | 886 | 3.7 | (3.5 - 3.9) | 483 | 3.9 | (3.6 - 4.3) | 403 | 3.4 | (3.1 - 3.8) |
| **2007** | 862 | 3.6 | (3.3 - 3.8) | 480 | 3.9 | (3.5 - 4.2) | 382 | 3.2 | (2.9 - 3.6) |
| **2008** | 889 | 3.7 | (3.4 - 3.9) | 481 | 3.9 | (3.5 - 4.2) | 408 | 3.4 | (3.1 - 3.8) |
| **2009** | 933 | 3.8 | (3.6 - 4.1) | 501 | 4.0 | (3.7 - 4.4) | 432 | 3.6 | (3.3 - 4.0) |
| **2010** | 722 | 3.0 | (2.8 - 3.2) | 386 | 3.1 | (2.8 - 3.4) | 336 | 2.8 | (2.5 - 3.1) |
| **2011** | 742 | 3.1 | (2.8 - 3.3) | 396 | 3.2 | (2.9 - 3.5) | 346 | 2.9 | (2.6 - 3.2) |
| **2012** | 643 | 2.7 | (2.5 - 2.9) | 358 | 2.9 | (2.6 - 3.2) | 285 | 2.4 | (2.1 - 2.7) |
| **2013** | 667 | 2.8 | (2.6 - 3.0) | 361 | 2.9 | (2.6 - 3.2) | 306 | 2.6 | (2.3 - 2.9) |
| **2014** | 676 | 2.8 | (2.6 - 3.0) | 350 | 2.9 | (2.6 - 3.2) | 326 | 2.8 | (2.5 - 3.1) |
| **2015** | 630 | 2.6 | (2.4 - 2.8) | 367 | 3.0 | (2.7 - 3.3) | 263 | 2.2 | (2.0 - 2.5) |
| **2016** | 654 | 2.7 | (2.5 - 2.9) | 371 | 3.0 | (2.7 - 3.3) | 283 | 2.4 | (2.1 - 2.7) |
| **2017** | 638 | 2.7 | (2.5 - 2.9) | 384 | 3.1 | (2.8 - 3.5) | 254 | 2.2 | (1.9 - 2.4) |
| **2018** | 605 | 2.5 | (2.3 - 2.7) | 339 | 2.8 | (2.5 - 3.1) | 266 | 2.3 | (2.0 - 2.6) |
| **2019** | 623 | 2.6 | (2.4 - 2.8) | 323 | 2.7 | (2.4 - 3.0) | 300 | 2.6 | (2.3 - 2.9) |
| **2020** | 481 | 2.1 | (1.9 - 2.2) | 266 | 2.2 | (2.0 - 2.5) | 215 | 1.9 | (1.6 - 2.1) |
| **Total** | **17229** | **3.3** | **(3.2 - 3.3)** | **9452** | **3.5** | **(3.5 - 3.6)** | **7777** | **3.0** | **(3.0 - 3.1)** |

**Supplementary Table I.** Overall and sex-stratified AAMR per 100,000 in Children Under 5 Years due to Pneumonia and Influenza in The United States, 1999-2020.
