# Supplementary material for: Emerging age, sex, ethnoracial, and regional trends in pneumonia and influenza-related mortality among children from 1999 to 2020
Source: Medicine (Baltimore). 2025 Apr 25;104(17):e42027. doi: 10.1097/MD.0000000000042027 (PMC12040052; doi:10.1097/MD.0000000000042027)
Supplement: Supplementary file 2 [file medi-104-e42027-s002.docx]

| **CRUDE MORTALITY RATE % CONFIDENCE INTERVAL** | | | | |
| --- | --- | --- | --- | --- |
| **YEAR** | **HISPANIC OR LATINO** | **ASIAN OR PACIFIC ISLANDER** | **BLACK OR AFRICAN AMERICAN** | **WHITE** |
| **1999** | 4.4 (3.8-5.0) | 4.8 (3.6 - 6.4) | 8.6 (7.7 - 9.5) | 3.5 (3.3 - 3.8) |
| **2000** | 4.2 (3.6-4.8) | 3.6 (2.5 - 4.9) | 7.5 (6.6 - 8.3) | 3.4 (3.1 - 3.7) |
| **2001** | 4.8 (4.2-5.4) | 2.3 (1.4 - 3.4) | 7.4 (6.5 - 8.2) | 3.6 (3.3 - 3.9) |
| **2002** | 3.7 (3.1-4.2) | 2.5 (1.7 - 3.7) | 7.0 (6.2 - 7.9) | 3.2 (2.9 - 3.4) |
| **2003** | 4.3 (3.8-4.9) | 3.8 (2.8 - 5.1) | 6.8 (6.0 - 7.7) | 3.7 (3.4 - 3.9) |
| **2004** | 3.7 (3.2-4.2) | 2.0 (1.3 - 3.0) | 7.0 (6.2 - 7.9) | 3.1 (2.9 - 3.4) |
| **2005** | 3.4 (2.9-3.9) | 2.8 (2.0 - 3.9) | 6.8 (6.0 - 7.7) | 2.8 (2.6 - 3.1) |
| **2006** | 3.6 (3.1-4.1) | 2.1 (1.4 - 3.0) | 6.6 (5.8 - 7.5) | 3.2 (2.9 - 3.4) |
| **2007** | 3.3 (2.9-3.8) | 2.6 (1.8 - 3.7) | 6.5 (5.7 - 7.3) | 3.0 (2.8 - 3.3) |
| **2008** | 3.6 (3.1-4.1) | 2.5 (1.7 - 3.5) | 6.4 (5.6 - 7.2) | 3.2 (2.9 - 3.4) |
| **2009** | 3.7 (3.2-4.2) | 2.2 (1.5 - 3.2) | 6.4 (5.7 - 7.2) | 3.3 (3.1 - 3.6) |
| **2010** | 2.8 (2.4-3.2) | 2.9 (2.1 - 4.0) | 4.9 (4.2 - 5.6) | 2.5 (2.3 - 2.7) |
| **2011** | 2.4 (2.1-2.8) | 2.0 (1.4 - 2.9) | 5.2 (4.5 - 5.9) | 2.7 (2.4 - 2.9) |
| **2012** | 2.3 (1.9-2.7) | 2.6 (1.8 - 3.5) | 4.5 (3.9 - 5.2) | 2.2 (2.0 - 2.5) |
| **2013** | 2.5 (2.1-2.9) | 1.8 (1.2 - 2.7) | 4.2 (3.6 - 4.8) | 2.5 (2.3 - 2.7) |
| **2014** | 2.3 (1.9-2.6) | 1.9 (1.3 - 2.8) | 5.6 (4.9 - 6.3) | 2.2 (2.0 - 2.5) |
| **2015** | 2.2 (1.8-2.5) | 2.3 (1.6 - 3.2) | 4.8 (4.1 - 5.4) | 2.1 (1.9 - 2.4) |
| **2016** | 2.3 (1.9-2.7) | 2.3 (1.6 - 3.2) | 5.3 (4.6 - 6.0) | 2.2 (2.0 - 2.4) |
| **2017** | 2.4 (2-2.8) | 2.3 (1.6 - 3.2) | 4.7 (4.0 - 5.4) | 2.2 (2.0 - 2.4) |
| **2018** | 2.1 (1.8-2.5) | 2.0 (1.4 - 2.8) | 4.0 (3.3 - 4.6) | 2.2 (2.0 - 2.5) |
| **2019** | 2.3 (1.9-2.6) | 1.9 (1.2 - 2.7) | 5.1 (4.4 - 5.8) | 2.1 (1.9 - 2.3) |
| **2020** | 1.6 (1.3-1.9) | 1.4 (0.9 - 2.1) | 3.5 (3.0 - 4.1) | 1.7 (1.5 - 1.9) |
| **Total** | **3.0 (2.9-3.1)** | **2.4 (2.2 - 2.6)** | **5.8 (5.6 - 6.0)** | **2.8 (2.7 - 2.8)** |

**Supplementary Table II.** Race-stratified AAMR per 100,000 in Children Under 5 Years due to Pneumonia and Influenza in The United States, 1999-2020
