# Supplementary material for: Emerging age, sex, ethnoracial, and regional trends in pneumonia and influenza-related mortality among children from 1999 to 2020
Source: Medicine (Baltimore). 2025 Apr 25;104(17):e42027. doi: 10.1097/MD.0000000000042027 (PMC12040052; doi:10.1097/MD.0000000000042027)
Supplement: Supplementary file 3 [file medi-104-e42027-s004.docx]

| **STATE** | **CRUDE RATE  (95% CI)** |
| --- | --- |
| **Connecticut** | 1.5 (1.2 - 1.9) |
| **Massachusetts** | 1.5 (1.3 - 1.8) |
| **Oregon** | 1.7 (1.4- 2.0) |
| **Rhode Island** | 1.8 (1.2 - 2.6) |
| **Maine** | 2.2 (1.6 - 3) |
| **New Jersey** | 2.2 (1.9 - 2.4) |
| **West Virginia** | 2.5 (1.9 - 3.2) |
| **New Hampshire** | 2.6 (1.9 - 3.4) |
| **Utah** | 2.6 (2.2 - 3) |
| **Idaho** | 2.7 (2.1 - 3.3) |
| **North Dakota** | 2.7 (1.8 - 3.8) |
| **Washington** | 2.7 (2.3 – 3.0) |
| **Colorado** | 2.8 (2.5 - 3.2) |
| **Iowa** | 2.8 (2.4 - 3.3) |
| **New York** | 2.8 (2.6 - 3) |
| **Virginia** | 2.8 (2.5-3.0) |
| **Alabama** | 2.9 (2.5-3.3) |
| **Maryland** | 2.9 (2.6-3.2) |
| **Kentucky** | 3.0 (2.6-3.4) |
| **Pennsylvania** | 3.0 (2.8-3.2) |
| **Vermont** | 3.0 (1.9-4.4) |
| **California** | 3.1 (2.9-3.2) |
| **Georgia** | 3.2 (3.0-3.5) |
| **North Carolina** | 3.2 (2.9-3.5) |
| **Delaware** | 3.3 (2.5-4.4) |
| **Missouri** | 3.3 (3.0-3.7) |
| **Texas** | 3.3 (3.2-3.5) |
| **Illinois** | 3.4 (3.2-3.7) |
| **Kansas** | 3.4 (2.9-3.9) |
| **Wisconsin** | 3.4 (3.1-3.8) |
| **Florida** | 3.5 (3.3-3.7) |
| **South Carolina** | 3.5 (3.1-3.9) |
| **Michigan** | 3.6 (3.4-3.9) |
| **Minnesota** | 3.6 (3.2-4.0) |
| **Montana** | 3.6 (2.7-4.7) |
| **Nebraska** | 3.7 (3.1-4.4) |
| **Ohio** | 3.7 (3.4-4.0) |
| **District of Columbia** | 4.0 (2.8-5.4) |
| **Nevada** | 4.1 (3.5-4.7) |
| **Tennessee** | 4.1 (3.7-4.5) |
| **Hawaii** | 4.3 (3.5-5.3) |
| **Indiana** | 4.4 (4.0-4.8) |
| **New Mexico** | 4.5 (3.8-5.2) |
| **Oklahoma** | 4.5 (4.0-5.0) |
| **Arizona** | 4.6 (4.2-5.0) |
| **Wyoming** | 4.6 (3.3-6.1) |
| **South Dakota** | 5.0 (4.0-6.3) |
| **Mississippi** | 5.2 (4.6-5.8) |
| **Arkansas** | 5.3 (4.7-5.9) |
| **Louisiana** | 5.7 (5.2-6.2) |
| **Alaska** | 6.9 (5.6-8.5) |

**Supplementary Table III.** State-stratified AAMR per 100,000 in Children Under 5 Years due to Pneumonia and Influenza in The United States, 1999-2020
